# Changes in endotracheal tube cuff pressure during laparoscopic surgery in head-up or head-down position

**DOI:** 10.1186/1471-2253-14-75

**Published:** 2014-08-31

**Authors:** Chun-Yu Wu, Yu-Chang Yeh, Ming-Chu Wang, Chia-Hsin Lai, Shou-Zen Fan

**Affiliations:** 1 Department of Anesthesiology, National Taiwan University Hospital, No.7, Zhongshan S. Rd. Zhongzheng Dist, Taipei City 10002, Taiwan

**Keywords:** Endotracheal tube cuff pressure, Laparoscopic surgery, Head-down position, Head-up position

## Abstract

**Background:**

The abdominal insufflation and surgical positioning in the laparoscopic surgery have been reported to result in an increase of airway pressure. However, associated effects on changes of endotracheal tube cuff pressure are not well established.

**Methods:**

70 patients undergoing elective laparoscopic colorectal tumor resection (head-down position, n = 38) and laparoscopic cholecystecomy (head-up position, n = 32) were enrolled and were compared to 15 patients undergoing elective open abdominal surgery. Changes of cuff and airway pressures before and after abdominal insufflation in supine position and after head-down or head-up positioning were analysed and compared.

**Results:**

There was no significant cuff and airway pressure changes during the first fifteen minutes in open abdominal surgery. After insufflation, the cuff pressure increased from 26 ± 3 to 32 ± 6 and 27 ± 3 to 33 ± 5 cmH_2_O in patients receiving laparoscopic cholecystecomy and laparoscopic colorectal tumor resection respectively (both *p* < 0.001). The head-down tilt further increased cuff pressure from 33 ± 5 to 35 ± 5 cmH_2_O (*p* < 0.001). There six patients undergoing colorectal tumor resection (18.8%) and eight patients undergoing cholecystecomy (21.1%) had a total increase of cuff pressure more than 10 cm H_2_O (18.8%). There was no significant correlation between increase of cuff pressure and either the patient's body mass index or the common range of intra-abdominal pressure (10-15 mmHg) used in laparoscopic surgery.

**Conclusions:**

An increase of endotracheal tube cuff pressure may occur during laparoscopic surgery especially in the head-down position.

## Background

Laparoscopic surgery is performed under general anesthesia with mechanical ventilation, and a high volume low pressure endotracheal tube with a sealing cuff pressure about 20 to 30 cmH_2_O is commonly used for a proper seal and avoidance of over-inflation [1-4].

There are several significant respiratory system changes during laparoscopic surgery. Abdominal CO_2_ insufflation elevates the intra-thoracic pressure [5,6] and adjusting patient positions by the head-up or head-down tilt results in a change in pulmonary compliance [7,8]. However, the impact of these physiologic alterations on endotracheal tube cuff pressure is not yet thoroughly clarified.

This study aimed to evaluate endotracheal cuff pressure changes during laparoscopic surgery after abdominal CO_2_ insufflation and positioning changes in head-up or head-down position. The correlations between airway pressure, body mass index (BMI), intra-abdominal pressure and cuff pressure change were also investigated.

## Methods

This prospective, observational study was conducted after obtaining approval from Research Ethics Committee of National Taiwan University Hospital (NTUH- 201107050RC). This study has been adhered to The Strengthening the Reporting of Observational Studies in Epidemiology (Additional file 1: STROBE) guidelines. After obtaining written informed consent in each patient, adult patients undergoing elective laparoscopic surgery in head-down and head-up position. Besides, patients undergoing elective open abdominal surgery in the supine position were enrolled as the control comparison. Exclusion criteria included the following: patients with a tracheostomy, a history of abnormal airway anatomy or lung disease with impaired compliance such as COPD.

All patients received general anaesthesia with fentanyl (1.5 ~ 2.5 mcg/kg), thiamylal (3 ~ 5 mg/kg), cisatracurium (0.15 ~ 0.20 mg/kg) and maintenance with 1.3 MAC of sevoflurane. The 1:1 ratio of air/oxygen mixture was used and nitrous oxide was not allowed in each patient. A low-pressure and high volume endotracheal tube (Kendall Curity®, Tyco Healthcare, Mansfield, MA, USA) was used in this study. The size of the endotracheal tube was 7.5 mm internal diameter for male and 7.0 mm internal diameter for female. Mechanical ventilation was used throughout the recording with a tidal volume of 8 to 10 ml.kg^-1^, and a rate between 8 and 14/min to maintain normocapnia without positive end-expiratory pressure use. The endotracheal tube cuff pressure was adjusted between 20 and 30 cm H_2_O by the manometer right before skin incision (VBM, Sulz, Germany) without leakage by stethoscopic ausculation [9]. In this study, each measurement was made with the same manometer but not multiple gauges. This manometer was calibrated monthly. This type of aneroid manometer is as accurate as a mercury manometer [10]. Before skin incision, a supplemental dose of muscle relaxant as a fifth of induction dose was given. During the induction and the whole investigation period, the train-of-four supramaximal twitch stimuli was set to ulnar nerve with a frequency of 2 Hz, four stimuli each separated by 0.5 s and repeated every 10 seconds. Each measurement was performed without observation of T1.

The endotracheal tube cuff pressure and airway pressure were measured during the intraoperative first 20 minutes and was recorded every 5 minutes in patients undergoing open abdominal surgery. Among patients receiving laparoscopic surgery, each patient received abdominal insufflation in the supine position with the intra-abdominal pressure kept between 10 to 15 mmHg. After abdominal insufflation, adjustments were made to patients in the head-up or head-down position (both 30°, measured using a protractor by an observer who did not participate in other parts of this study) and consequent cuff pressure and airway pressure changes were recorded during end-expiration. Measurement was taken with each patient's head and neck in the neutral position and the occiput on a same type of pillow and data was recorded 2 minutes after abdominal insufflation and positioning adjustment.

Data analysis was performed using SigmaPlot for Windows version 12 (SAS Institute, Cary, NC, USA). This study fit a power of 80% and a *p* value of < 0.05 was considered to be statistically significant. A number of ten patients in each group was calculated to detect a mean cuff pressure change by 5 cmH_2_O. The one-way repeated measures ANOVA test and post hoc analysis with the Tukey method were used to compare changes of cuff pressure after CO_2_ insufflation and patient positioning changes. The Pearson correlations were calculated to determine the relationships of airway pressure, BMI, intra-abdominal pressure to the change in endotracheal tube cuff pressure after abdominal CO_2_ insufflation and positioning.

## Results

Baseline characteristics of 15 patients undergoing open abdominal surgery and 70 patients undergoing laparoscopic surgery are presented in Table 1 (all p > 0.05). There are 15 patients receiving open abdominal surgery (10 for colorectal tumor resection and 5 for hysterectomy), 32 patients receiving laparoscopic cholecystectomy (head-up position) and 38 patients receiving laparoscopic colorectal tumor resection (head-down position). Among patients undergoing laparoscopic cholecystectomy, the measurement took 7 ± 2 minutes. Among patients undergoing laparoscopic colorectal tumor resection, the measurement took 7 ± 3 minutes.

**Table 1:** Characteristics of 15 patients scheduled for open abdominal surgery in supine position and 70 patients (32 in the head-up and 38 in the head-down position) scheduled for laparoscopic surgery

	**Open surgery**	**Head-up**	**Head-down**
	**(N = 15)**	**(N = 32)**	**(N = 38)**
**Age (year)**	50.0 ± 11.3	56.4 ± 16.9	50.2 ± 17.1
**Sex (F/M)**	6/9	19/13	20/18
**Body height (cm)**	160.3 ± 7.5	160.5 ± 9.2	160.4 ± 7.5
**Body weight (kg)**	60.1 ± 118	66.6 ± 13.4	61.3 ± 9.6
**BMI (kg.m**^**-2**^**)**	23.2 ± 2.8	25.9 ± 4.6	23.8 ± 3.6
**IAP (mmHg)**	NA	13.4 ± 1.5	13.1 ± 1.5

Body mass index: body mass index; IAP: intra-abdominal pressure.

Values are mean (SD) or number (proportion).

### Cuff pressure and airway pressure changes in patients undergoing open abdominal surgery as control comparison

Among patients undergoing open abdominal surgery, there was a slight decrease of cuff pressure at the 20st minutes of surgery but no significant cuff pressure changes during the first 15 minutes (mean cuff pressure: 24 ± 3 cmH_2_O v.s. 22 ± 4 cmH_2_O, at 0 minutes and the 20th minutes respectively, p < 0.05). There was no significant airway pressure change during the investigating period in patients undergoing open abdominal surgery (p = 0.108).

### Effects of abdominal CO_2_ insufflation

Among patient undergoing laparoscopic surgery, after abdominal CO2 insufflation with the study subjects in the supine position, the mean cuff pressures increased from baseline values of 27 ± 3 and 26 ± 3 to 33 ± 5 and 32 ± 6 cmH_2_O (both p < 0.001), for patients undergoing laparoscopic colorectal tumor resection surgery (head-down group) and laparoscopic cholecystectomy (head-up group), respectively. The mean airway pressure also increased significantly from the supine baseline value of from 18 ± 4 to 25 ± 4 cmH_2_O and 19 ± 4 to 26 ± 6 cmH_2_O (both *p* < 0.001), for patients undergoing laparoscopic colorectal tumor resection surgery (head-down group) and laparoscopic cholecystectomy (head-up group) respectively. There was no patient with the cuff pressure decreased after abdominal insufflation and the highest increase in cuff pressure was 20 cmH_2_O.

### Effects of positioning changes

For patients undergoing laparoscopic colorectal tumor resection surgery, the head-down position led to slightly increases in mean cuff pressure and mean airway pressure from 33 ± 5 to 35 ± 5 and from 25 ± 4 to 26 ± 5 cmH_2_O respectively (both *p* < 0.001). For patients undergoing laparoscopic cholecystectomy, the head-up position, resulted no significant mean cuff pressure and mean airway pressure changes (mean cuff pressure from 32 ± 6 to 32 ± 6 cmH_2_O and mean airway pressure from 26 ± 6 to 25 ± 6 cmH_2_O respectively, both *p* > 0.05). The increase of mean cuff pressure resulting from the head-down tilt was much lower than that caused by the CO_2_ insufflation (2 ± 2 versus 6 ± 3 cmH_2_O).

There were 6 patients undergoing laparoscopic colorectal tumor resection that had a total increase of cuff pressure more than 10 cm H_2_O (18.8% of the group) with a highest increase of 20 cm H_2_O. There were 8 patients undergoing laparoscopic cholecystectomy had a total increase of cuff pressure more than 10 cm H_2_O (21.1% of the group) with a highest increase of 18 cm H_2_O.

### Correlations of airway pressure change, BMI, intra-abdominal pressure to endotracheal tube cuff pressure

There were correlations between changes of airway pressure and endotracheal tube cuff pressure by abdominal insufflation (r = 0.68, *p* < 0.05, Table 2). The changes of airway pressure and endotracheal tube cuff pressure by positioning were not correlated.

**Table 2:** Correlation coefficient of airway pressure change, body mass index, intra-abdominal pressure to endotracheal tube cuff pressure change after insufflation and position changes

**correlation coefficient (r)**	**CO2 insufflation effects**	**Positioning effects**	
		**Head-down**	**Head-up**
	**(N = 70)**	**(N = 38)**	**(N = 32)**
**Airway pressure change**	0.68^*^	0.03	0.34
**Body mass index**	0.23	0.02	-0.28
**Intra-abdominal pressure**	-0.05	-0.26	-0.07

^*^p < 0.05.

**Figure 1:**
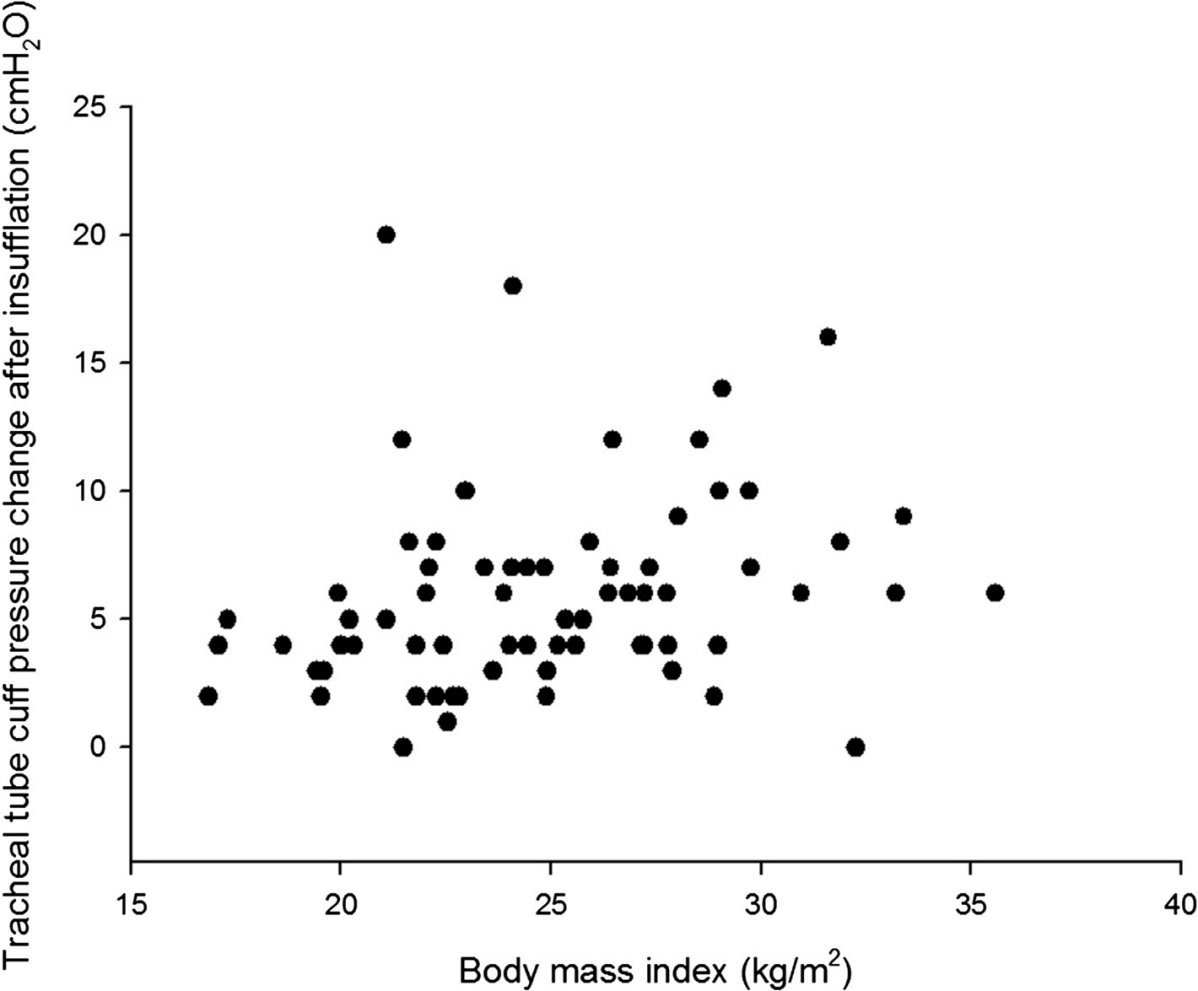
**Scatterplot of cuff pressure changes after CO**_**2 **_**insufflation and body mass index.**

**Figure 2:**
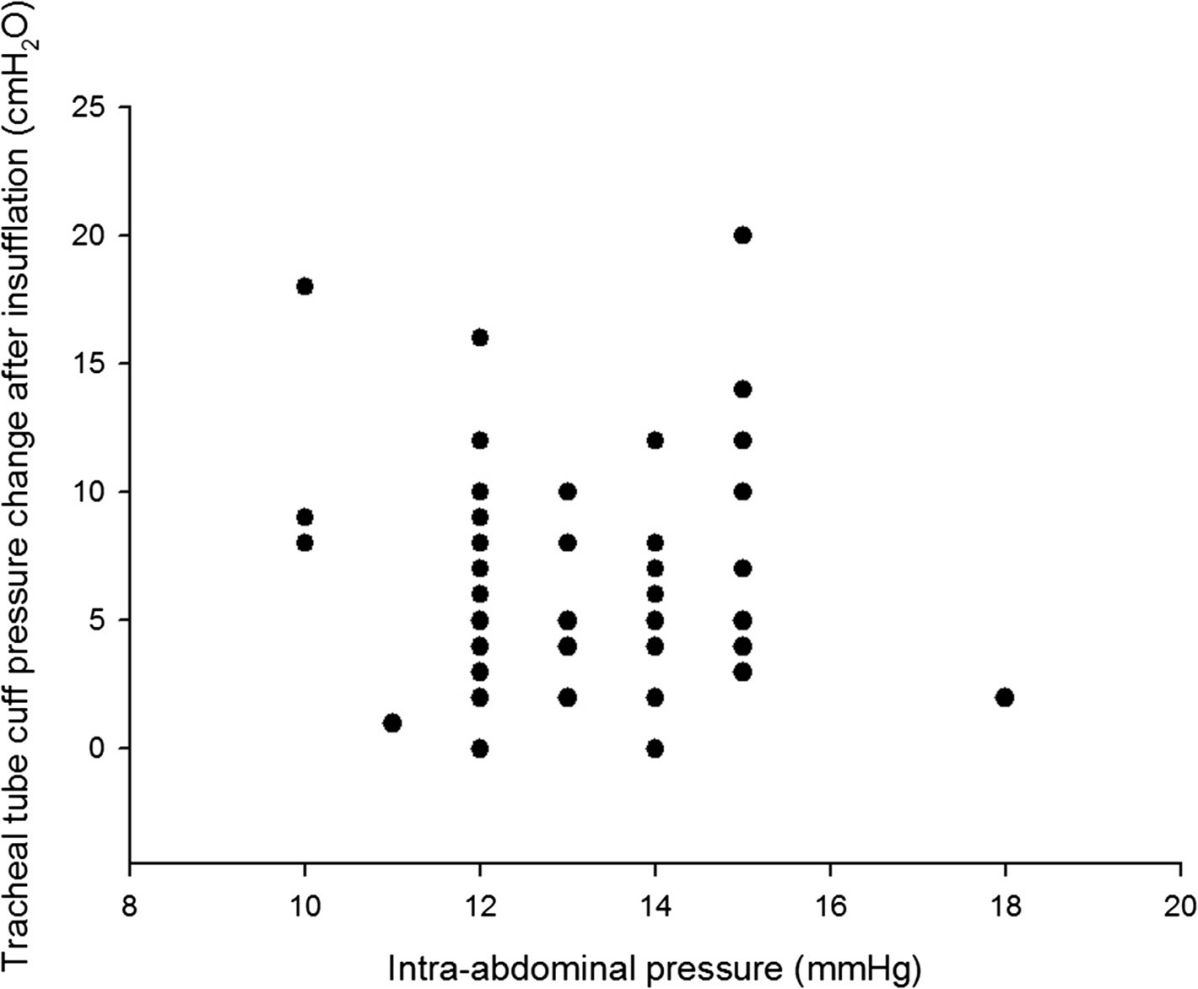
**Scatterplot of cuff pressure changes after CO**_**2 **_**insufflation and intra-abdominal pressures.**

The range of BMIs in our patients was between16.85 and 35.58 kg.m^-2^. There were no significant correlations between neither BMI nor intra-abdominal pressure and neither changes of endotracheal tube cuff pressure by abdominal insufflation nor by positioning in both groups (all *p* > 0.05, Table 2). The patient with the highest increase in cuff pressure of 20cmH_2_O after abdominal insufflaiton had a BMI of 21.08 kg.m^-2^. The scatterplots of BMI, intra-abdominal pressure and cuff pressure changes after CO_2_ insufflation were presented in Figures 1 and 2.

## Discussion

The major finding of our study is that the endotracheal tube cuff pressure increases with abdominal insufflation and patient position changes, especially the head-down position, during laparoscopic colorectal tumor resection surgery.

Maintenance of adequate endotracheal tube cuff pressure is not only important to avoid ventilatory leak during mechanical ventilation but also important to prevent aspiration especially for patients in head-down position [11,12]. Conversely, several postoperative complications such as such as cough, sore throat, hoarseness and blood-streaked expectorations are associated with excessive endotracheal tube cuff pressure [13,14].

The present study indicated only a modest mean increase of cuff pressure, but there were also substantial proportions of patients (about 20% in both groups) with increases of cuff pressures more than 10 cm H_2_O. Besides, a modest mean increase of cuff pressure still may be clinical relevant especially in vulnerable patients. Calder et al had reported that the incidence of postoperative sore throat increased with cuff pressure in paediatric day-case surgery with an average anaesthesia time of 60 minutes. The incidences of sore throat by cuff pressures in their study were 4% at 11–20 cmH_2_O, 20% at 21–30 cmH_2_O, 68% at 31–40 cmH_2_O, and 96% at >40 cmH_2_O [15]. The incidence of sore throat was abruptly elevated in cuff pressure above 30 cmH_2_O. Because the laparoscopic surgery generally took longer time than the day-case surgery, the increase of endotracheal tube cuff pressure during the laparoscopic surgery may raise risks of postoperative complications. Therefore, re-adjustment of cuff pressure to between 20 to 30 cm H_2_O after CO_2_ insufflation and positioning may be warranted in each patient.

Yildirim et al had found the cuff pressure elevation and a higher incidence of postoperative sore throat in the laparoscopic than open cholecystectomy that was compatible to our findings [16]. The differences between their report and our findings are that they did not analyze the position effect and correlations of cuff pressure to body mass index and intra-abdominal pressure. Recently, Lizy et al reported that cuff pressure increased by changes in patients’ positioning in critically ill patients treated with mechanical ventilation [17]. Our finding regarding the head-down position effect on cuff pressure was compatible to their finding. Additionally, we found that head-up position effect was less relevant during the laparoscopic surgery.

In this report, we only investigated a short period of time which involved most interested confounding factors (abdominal insufflation, positioning) during the laparoscopic surgery. During the surgery, cuff pressure may vary due to temperature changes or gas diffusion over time. Gas diffusion to cuff was not likely the reason of increased cuff pressure because nitrous oxide was not used. A slight decrease of cuff pressure was noted at the 20st minute in patients undergoing open abdominal surgery. It may be due to repetitive measurement resulting a slow leak of air in the cuff from the manometer. However, during the first 15 minutes, the cuff pressure did not significantly change and the measurement period in patients undergoing laparoscopic surgery was much shorter (about 7 minutes). Therefore, it was not likely that an increase of cuff pressure after abdominal insufflation was the normal characteristic of intraoperative variation.

There were reports indicating endotracheal shortening in the laparoscopic surgery with cephalad movement of the carina [18,19] and the conformational change of the trachea by abdominal insufflation and the gravitational effect of the head-down tilt may be the underlying mechanism behind the elevated cuff pressure. In the present study, an excessive cuff pressure during laparoscopic surgery was found not only in obese but also non-obese patients. Besides, our data showed that the patient with the greatest cuff pressure increase has a BMI below the general mean value. An intra-abdominal pressure between 10 to 15 mmHg is considered clinically well tolerated and necessary for adequate visualization and exposure of the operative field [8,20]. In our data, no significant correlation was found between intra-abdominal pressure ranging from 10 to 15 mmHg and cuff pressure changes. Therefore, excessive endotracheal tube cuff pressure in the laparoscopic surgery may not be preventable with a CO_2_ insufflation pressure at the lower limit of common intra-abdominal pressure usage.

There are several limitations in this report. Firstly, this study is limited by the non-blinding nature and accuracy of measurement technology. Although this type of manometer used in this study is one of the most convenient and common tool in literatures, the set and measured endotracheal tube cuff pressures are only accurate to within 1 cmH_2_O. Smaller variation of cuff pressure can't be detected by this device but it may be less clinical relevant. Secondly, the study design was not able to correlate cuff pressure and specific clinical outcome such as postoperative sore throat, that was associated with multiple factors, such as numbers of attempt to intubate, use or no use of a intubating stylet, operating time, type of lubrication of endotracheal tube cuff…etc. Each factor should be considered and analyzed to clarify between the specific factor- cuff pressure and postoperative sore throat. Our study may be a potential reference to further studies. Thirdly, our result may not be extrapolated to general laparoscopic surgery until future data collected during multiple types of laparoscopic procedures is available.

## Conclusion

On the basis of our result, we can suggest an inadvertent increase endotracheal tube cuff pressure may be found in some kinds of laparoscopic surgery especially in colorectal tumor resection in head-down position. The increase of cuff pressure may not be associated with body mass index and the common range of intra-abdominal pressure (10 to 15 mmHg) during the laparoscopic surgery.

## Competing interests

The authors declare that they have no competing interests.

## Authors’ contributions

CYW participated in the study design and manuscript writing. YCY participated in patient enrolment. CHL carried out the data collection. MCW carried out data analysis. SZF participated in the study design. All authors read and approved the final manuscript.

## Pre-publication history

The pre-publication history for this paper can be accessed here:

http://www.biomedcentral.com/1471-2253/14/75/prepub

## Supplementary Material

Additional file 1 STROBE Statement—checklist of items that should be included in reports of observational studies. Click here for file
